# Welcome to *Herpesviridae *-- a new premier virology journal

**DOI:** 10.1186/2042-4280-1-1

**Published:** 2010-12-07

**Authors:** Mensur Dzabic, Robert Hendricks, Christian Münz, Cecilia Söderberg-Nauclér

**Affiliations:** 1 Department of Medicine, Karolinska Institutet, Stockholm, Sweden; 2 Department of Ophthalmology, University of Pittsburgh School of Medicine, Pittsburgh, PA, USA; 3 Department of Immunology, University of Pittsburgh School of Medicine, Pittsburgh, PA, USA; 4 Department of Microbiology and Molecular Genetics, University of Pittsburgh School of Medicine, Pittsburgh, PA, USA; 5 Viral Immunobiology, Institute of Experimental Immunology, University of Zürich, Zürich, Switzerland

## Editorial

The ability to establish lifelong latency after primary infection is a common denominator of large DNA viruses of the Herpesviridae family. Herpes viruses are endemic to humans worldwide and contribute to the meta-genome of 70-100% of the world's population. The significance of herpes viruses as pathogens has been increasingly recognized during the past few decades, with the escalation in the number of patients undergoing immunosuppressive therapy after organ or bone marrow transplantation, and the rising incidence of AIDS. The ability of these viruses to ensure their survival by controlling cellular and immunological functions [1] has focused attention on their potential role in the development of vascular [2] and autoimmune syndromes [3], cancers [4], and as possible co-factors for acquisition of HIV [5]. In addition, these viruses have co-evolved with, and shaped, the immune systems of their hosts. The secrets of immunology can only be unlocked efficiently by using well-adapted pathogens like these as probes or keys to reveal fine adjustments in the immune system compartments that have developed to control these promiscuous and potentially deadly pathogens [6].

A search in PubMed [7] reveals that approximately 2000 papers focusing on herpes viruses are published each year, many in well-established general virology journals such as *Journal of Virology *and *Virology*. However, many others are published in specialist journals focusing on immunology, pathology, transplantation, and other areas. We aim for *Herpesviridae *to be our community's premier journal, bringing together the interdisciplinary niches of research on this important virus family Our goal is to focus the efforts of many specialities into one journal by providing an Open Access platform to gather and advance our scientific efforts. *Herpesviridae *is in its infancy and is therefore very flexible. In close co-operation with you -- our colleagues in the field -- we hope to develop it into a journal that we would all like to publish in and read. For that reason, your input is vital for our ability to continue improving the journal. All input will be considered carefully, and we will reply in a timely manner.

One of the major differences with *Herpesviridae *is the focus on you as the author and how you can best publish your scientific work. Together with our internationally well-renowned Editorial Board [8], consisting of today's and tomorrow's leaders in our field, we will apply a stringent but rapid peer-review process, with the goal of giving authors a first decision within three weeks after submission. *Herpesviridae *uses a closed peer review system in which articles are reviewed by at least two experts selected by the assigned Editors-in-Chief, after an initial screening for suitability. With rapid publication in mind, we will weight the reviews that we receive and prioritize the concerns raised, so that revision time and effort can be focused on the most pressing improvements.

*Herpesviridae *is an online publication only. Thus, articles will not be restricted in size, will be available immediately after acceptance, and will be listed shortly thereafter in PubMed. Furthermore, *Herpesviridae *is an Open Access journal, enabling universal accessibility and ensuring that your work reaches the widest possible audience [9]. Authors will also retain the copyright for their work. Universal accessibility, together with the fact that *Herpesviridae *will publish high quality articles that have undergone stringent peer review, will hopefully result in high citations for published articles and consequently a high Impact Factor in the future.

*Herpesviridae *publishes articles contributing to overall knowledge on the role of herpes viruses in health and disease. Articles from diverse fields, from experimental biological studies to clinical studies, are welcome. We aim to publish high-quality Reviews, Research articles, Short Reports, Editorials, Letters to the Editor, Case Reports, Clinical studies, and Meeting Reports of special interest. The articles will be deposited in freely accessible, full-text repositories such as PubMed Central in accordance with the policies of many major funding agencies [10-13].

Alongside, all the advantages of *Herpesviridae*, the key to the success of this journal ultimately rests with you, our authors, reviewers, and readers. We are very excited about *Herpesviridae *and the opportunities that it provides to our field. We hope you will seize the opportunity to contribute and develop this new journal. We welcome your comments, advice and submissions.
